# Study on the expression profile and role of decorin in the progression of pancreatic cancer

**DOI:** 10.18632/aging.203060

**Published:** 2021-05-21

**Authors:** Litao Zhang, Chao Liu, Huijie Gao, Caiju Zhou, Wei Qin, Jian Wang, Lingxin Meng, Huiyun Wang, Qiang Ren, Yuntao Zhang

**Affiliations:** 1 Department of Biological Science, Jining Medical University, Rizhao, Shandong, China; 2 Department of Pharmacy, Jining Medical University, Rizhao, Shandong, China; 3 Department of Pancreatic Oncology, Tianjin Cancer Institute and Hospital, Tianjin Medical University, Tianjin, China; 4 Department of Oncology, People's Hospital of Rizhao, Shandong, China

**Keywords:** decorin, expression profile, pancreatic cancer, isoforms

## Abstract

Desmoplasia in the extracellular matrix (ECM) is one of the hallmarks of pancreatic cancer (PC), a virtually incurable disease. Decorin, a classical small leucine-rich proteoglycan found in the ECM, was upregulated in PC tissue samples according to the data of TCGA. However, decorin plays a protective role in the ECM. So it is necessary to study the roles of decorin in the progression of PC. A significantly upregulated expression of decorin was observed in the PC tissue samples compared with the normal tissues. However, there was no considerable difference in the level of expression of decorin during different pathological stages, which was supported by the immunoblot analysis. Western blot showed a higher expression of decorin A in the para-carcinoma tissue than in the cancerous tissue but the expression of decorin B, C, and D was elevated in the cancerous tissue. The results of the MTT and scratch wound healing assays revealed an elevated proliferation ability and migration rate in decorin B-overexpressing cells but were inhibited in the decorin A-overexpressing cells. Overexpression of decorin A significantly elevated the expression of the apoptosis-related genes and Decorin B-overexpression elevated proliferation-related genes. All the results showed that decorin B played important roles in the promoting of PC.

## INTRODUCTION

Over the past few decades, there has not been a significant improvement in the prognosis of pancreatic cancer (PC); thus, it is still considered a virtually incurable disease [1]. PC is the fourth-leading cause of cancer-related fatalities in men and women, with an overall 5-year survival rate of 9% [2]. Most PC patients remain asymptomatic until the disease reaches an advanced stage [3]. Despite extensive research on PC, the mechanism involved in controlling tumorigenicity of PC is unclear.

In solid tissues, the extracellular matrix (ECM) comprising polysaccharides, water, and proteins provides structural support to the cells [4, 5]. The ECM is highly dysregulated and remodeled in several diseases, especially cancers [6, 7]. Excessive desmoplasia in the ECM is the trademark of PC, which contributes to its aggressive behavior [8], and chemoresistance through tumor-stromal interactions [9]. Thus, it is necessary to study the interaction between the ECM, tumor formation, and invasion/metastasis in PC.

Small leucine-rich proteoglycans (SLRPs) are a subgroup of proteoglycans that organize and regulate cell development in the ECM. Based on their chromosomal organization, evolutionary conservation, N-terminal cysteine-rich clusters, and leucine-rich repeats (LRR), the ubiquitous SLRPs are divided into five distinct classes [10, 11]. Decorin, a class I SLRP, is a 40 kDa pan-tyrosine kinase inhibitor containing a single dermatan sulfate (DS)/chondroitin sulfate (CS) chain bound to the N-terminal of core protein [12]. In the genbank of NCBI, there are 6 transcripts: *decorin A* has 2 transcripts with the full-length of 6850 bp and 6805 bp, respectively and encodes the same isoform-decorin A, with 359 amino acids. *Decorin B, C, D, E* all have 1 transcript with the full-length of 3643 bp, 3529 bp, 3409 bp and 3296 bp, respectively, coding decorin B, C, D, E consist of 250, 212,172 and 75 amino acids. LRRNT (leucine rich repeat N-terminal domain) and LRR motif exist in the decorin isoforms, but number of LRR motif is different, 10 for decorin A, 6 for decorin B, 4 for decorin C, 2 for decorin D and 0 for decorin E. Nowadays. Most studies were concentrated on decorin A. It binds to receptor tyrosine kinases, leading to receptor dimerization and subsequent phosphorylation. This is followed by the activation of mitogen associated protein kinase (MAPK) pathway, followed by p21 expression resulting in cell cycle inhibition [13]. Alternatively, decorin blocks angiogenic growth signals to restrict tumor growth and dissemination [14]. Thus, decorin plays a protective role in the ECM. Previous studies showed downregulated expression of decorin in invasive breast cancer [15], urothelial cancer [16], colon cancer [17], non-small cell lung cancer [18], esophageal squamous cell cancer [19], prostate cancer, [20] etc. Conversely, its expression was found to be elevated in PC [21]. Meanwhile, there are very few studies about other decorin isoforms. Thus, it was necessary to study the expression profile and function of isoforms decorin in PC progression.

Here, first, we determined the expression of decorin in PC patients, followed by the analysis of the decorin isoforms, finally, the decorin isoforms function study in the cell levels will be conducted. The results of this study would help us understand the mechanism of decorin expression in PC progression.

## RESULTS

### Decorin expression profile in PC

**Figure 1:**
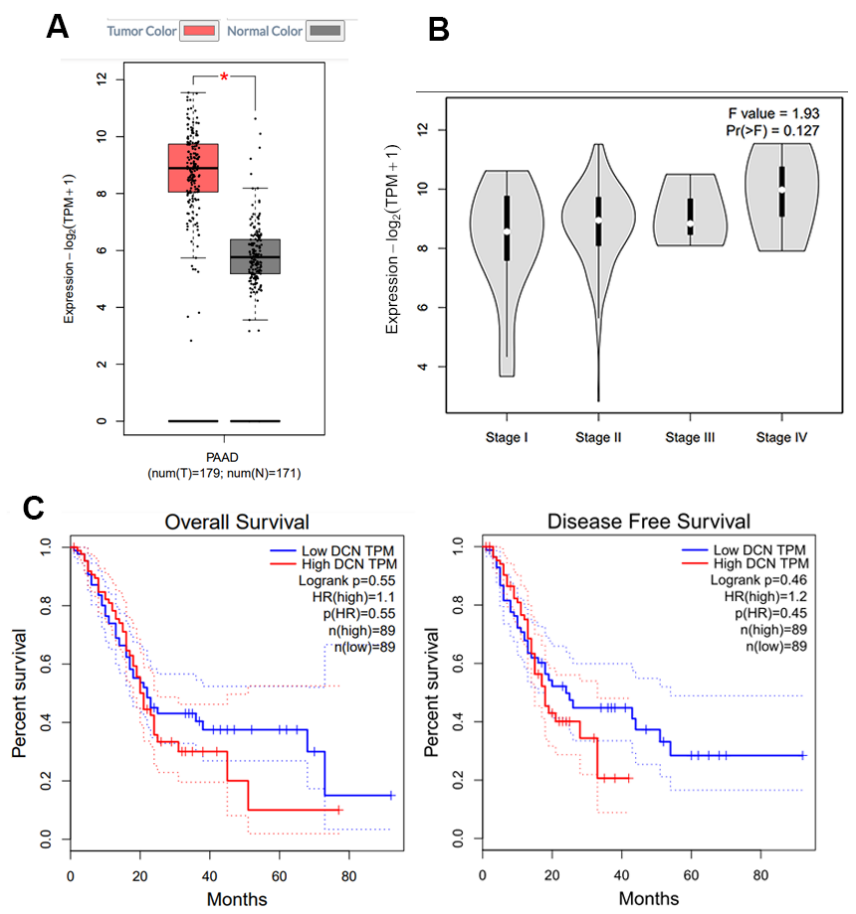
**Decorin expression in PC by GEPIA.** (**A**) Expression profile of decorin in 350 PC cases (tumor: 179; control: 171), *p<0.05; (**B**) The correlation between decorin expression and the pathological stage in PC (GEPIA). (**C**) Overall and disease-free survival analysis of decorin expression in PC (n = 178).

GEPIA was used to study decorin expression using boxplots, pathological stage plots, and survival curves (Figure 1A–1C). We observed a significantly upregulated expression of decorin in PC tissue samples compared with the normal tissues (Figure 1A, Log2FC < 2, *p*-value < 0.01). However, there was no significant difference during different pathological stages (Figure 1B). Moreover, during the early stages of PC, patients with low levels and high levels of decorin had a similar overall survival rate and disease-free survival rate; however, the difference in survival rate became significant during advanced stages of the disease.

**Figure 2:**
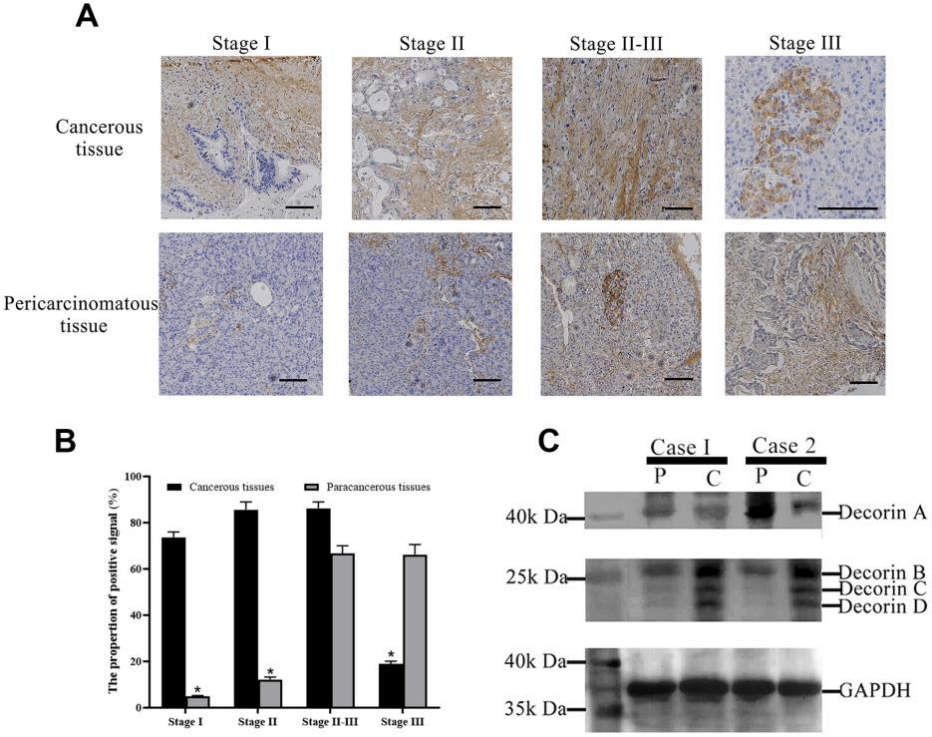
**Decorin expression profile in PC patients.** (**A**) Immunohistochemical analysis of decorin during the pathological stages of PC, bar indicates 100 μm; (**B**) The quantification of decorin during the pathological stages of PC based on the results of immunohistochemical analysis. (**C**) Western blot of decorin in cancerous and para-cancerous tissue of PC patients, C: cancerous tissues, P: paracancerous tissues.

Immunohistochemistry was done to evaluate the expression of decorin including all the isoforms in both cancerous and para-cancerous tissue of patients with PC by polyclonal antibody of decorin. The results showed that decorin had wider distribution and higher expression in cancerous tissue than in para-cancerous tissue during stage I-II of the disease. In the stage II-III, distribution and expression of decorin were similar in both the cancerous and the para-cancerous tissue. In stage III, the expression and distribution became more apparent in the para-cancerous tissue (Figure 2A). Image J was used to quantify the signal of decorin and the results were shown in Figure 2B to further support our analysis based on Figure 2A. Western blot showed a higher expression of decorin A in the para-cancerous tissue than in the cancerous tissue, but decorin B, C, and D expressions were elevated in the cancerous tissue (Figure 2C).

### Effect of decorin on BxPC-3 cells

**Figure 3:**
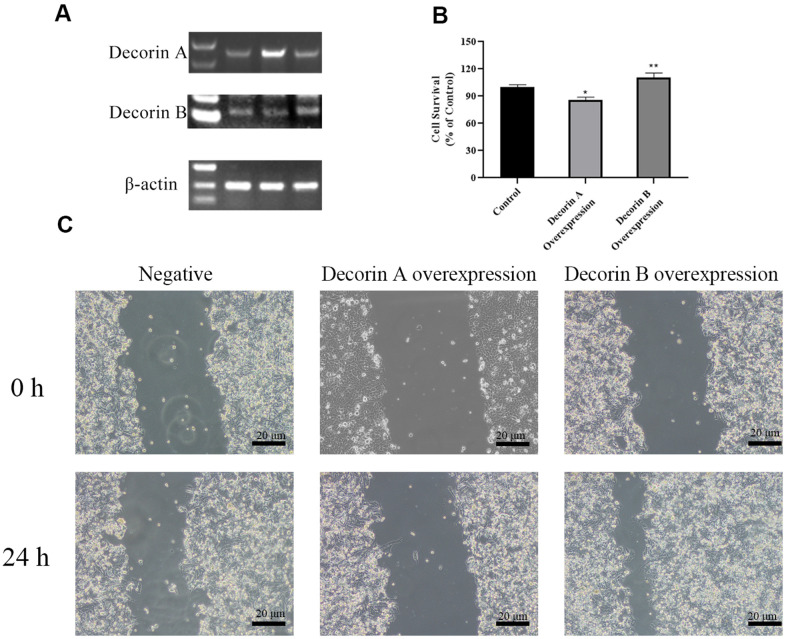
**The effects of decorin A and decorin B on the BxPC-3 cells.** (**A**) Semi-quantitative RT PCR analysis for decorin A and B after transfected (**B**); Cell viability analysis by MTT assay after the overexpression of decorin A and B; (**C**) Cell migration analysis by the scratch wound assay after the overexpression of decorin A and B.

We overexpressed decorin A and decorin B in BxPC-3 cells. Semi-quantitative RT PCR was used to confirm the over-expression of decorin A and decorin B (Figure 3A). The results of the MTT assay showed that cell proliferation was elevated in decorin B-overexpressing cells but inhibited in decorin A-overexpressing cells (Figure 3B). Next, we evaluated the influence of decorin A and B on the migration of BxPC-3 cells. The results demonstrated that PC cell migration was enhanced by the overexpression of decorin B and inhibited by the overexpression of decorin A (Figure 3C).

### The effect of decorin A and B on the expression of proliferation- and apoptosis-related genes

**Figure 4:**
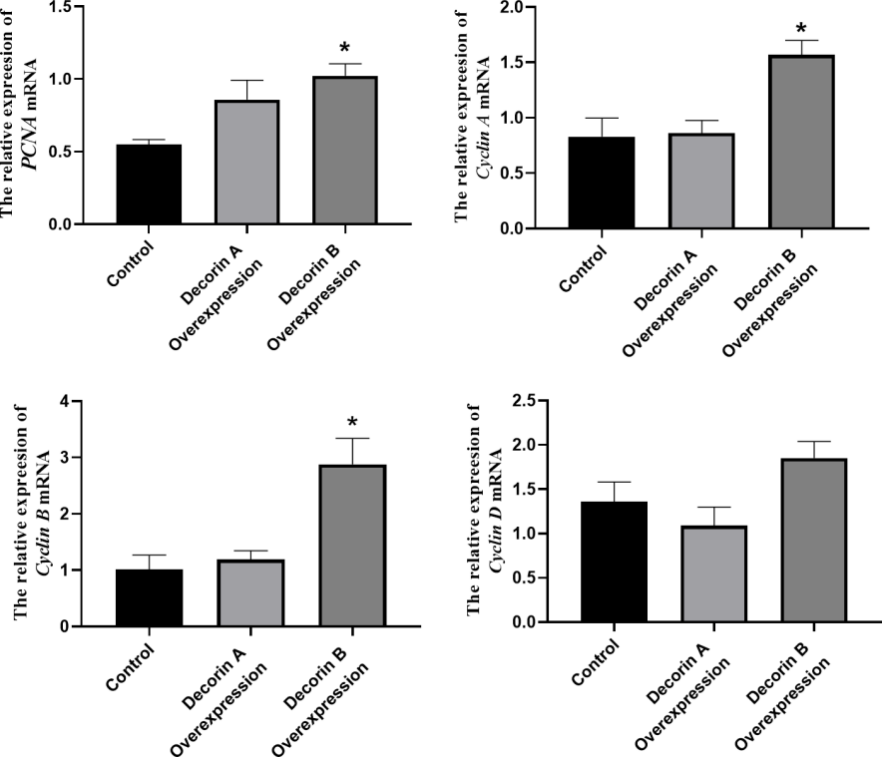
The effect of decorin A and B on the expression of proliferation-related genes in BxPC-3 cells.

Decorin usually binds to receptor tyrosine kinases, leading to receptor dimerization and subsequent phosphorylation, which then affects p21 pathway [13]. In the p21 pathway, PCNA, cyclin A, cyclin B, and cyclin D are important molecular for cell proliferation while BCL2, BAX, and p53 for cell apoapsis. For the representative genes involving in cell proliferation including PCNA, cyclin A, cyclin B, and cyclin D, there was no substantial difference between the control and decorin A-overexpressed group (Figure 4). However, a significantly elevated expression of PCNA, cyclin A, and cyclin B was observed in the decorin B-overexpressed group compared with the control group.

**Figure 5:**
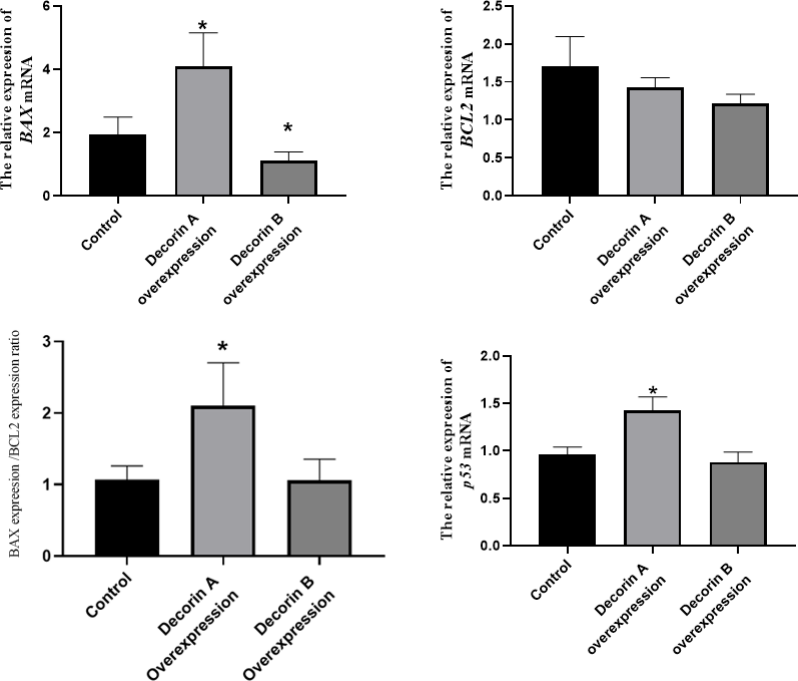
The effect of decorin A and B on the expression of apoptosis-related genes in BxPC-3 cells.

There was a significant increase in the expressions of BAX and p53 in the decorin A-overexpressed group and there was a considerable reduction in the expression of BAX in the decorin B-overexpressed group than the control group (Figure 5).

## DISCUSSION

The process of cancer progression involves silencing the expression of tumor suppressor genes and activating mutations in oncogenes, conferring selective advantages to basic cellular processes, including cell migration, proliferation, and metastasis. Recent studies have demonstrated the vital role played by the surrounding tumor stroma in tumor growth and progression, including aberrant synthesis and proteoglycan deposition [12]. Decorin, a member of the SLRP family, is known to play a regulatory role in ECM formation [22]. Decorin sequesters several growth factors, such as transforming growth factor (TGF)-β1 and directly inhibits several members of the receptor tyrosine kinase (RTK) family, including the insulin-like growth factor receptor I (IGF-IR), the epidermal growth factor receptor (EGFR), and the hepatocyte growth factor receptor (Met), inducing potent tumor repression [23]. Thus, decorin is usually recognized as an anti-cancer gene. On the contrary, in this study, we observed a higher expression of decorin in PC samples (Figures 1A, 2A). Thus, we wanted to explore why was the anti-cancer gene, decorin, expressed highly in PC. The western blot results showed that decorin A (the complete decorin) demonstrated lower expression in the cancerous tissue than in the para-cancerous tissue while decorin B, C, and D (the decorin isoforms) showed higher expression (Figure 2B), which was probably the main reason for the high expression of decorin in PC. Thus, we hypothesized that decorin A, as the anti-cancer gene, showed lower expression, but its isoforms might promote tumor progression in PC. Next, we overexpressed decorin A and B in BxPC-3 cells to analyze the effects of overexpression.

**Figure 6:**
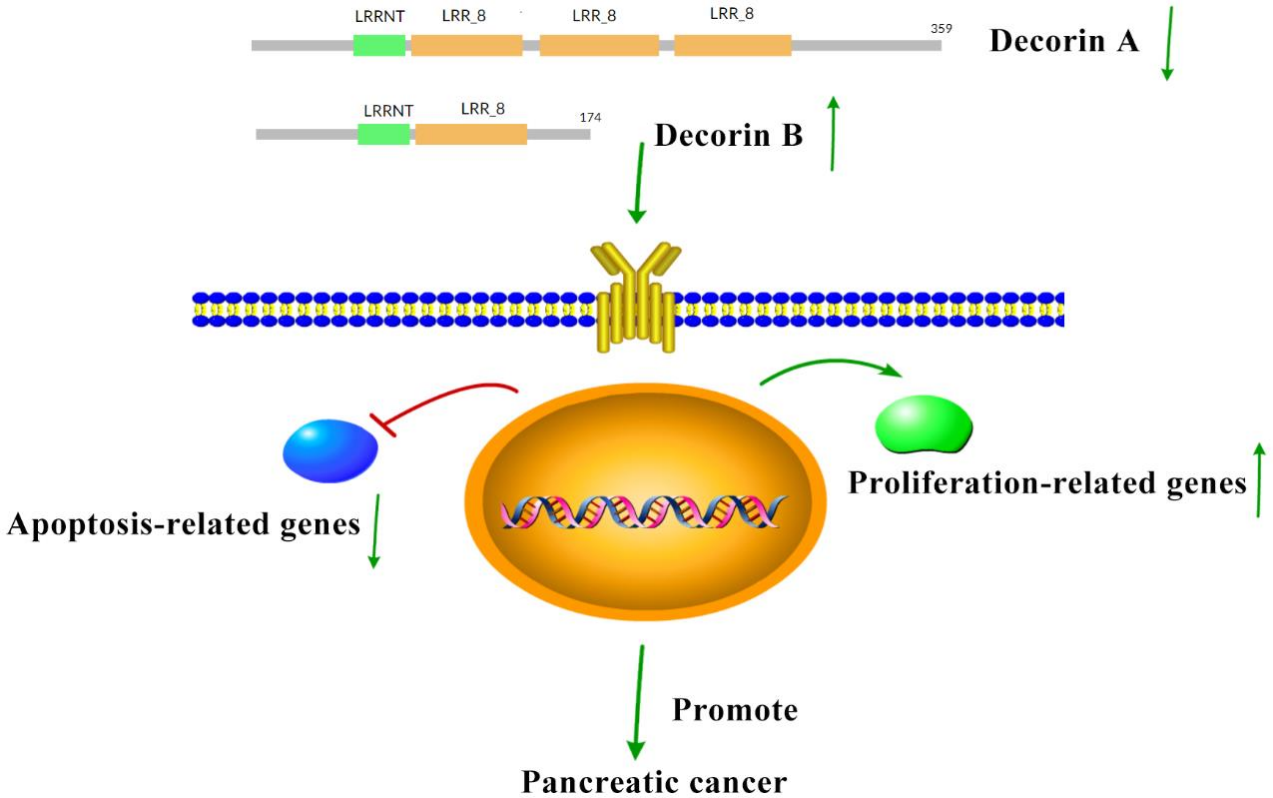
**The hypothesis for the mechanism of decorin in promoting PC.** In PC, the expression of decorin A decreases while decorin B increases, which can inhibit apoptosis and enhance proliferation to promote PC progression.

The overexpression of decorin A inhibits cell migration and proliferation in BxPC-3 cells (Figure 2), which was consistent with the results in other cancer cell lines [24]. Meanwhile, the cell proliferation-related genes—PCNA, cyclin A, cyclin B, and cyclin D—were not affected by the overexpression of decorin A but the expression of apoptosis-related genes—p53 and BAX—was upregulated (Figure 3). A previous study showed that decorin interacted with TGF-β and TGF-β signaling played a critical role in regulating cell migration [25, 26]. However, our results showed that decorin A overexpression did not affect TGF-β signaling as there was no significant difference in the expression of proliferation-related genes. In U87MG glioma cells, decorin-mediated inhibition of cell migration involved the activation of autophagy and suppression of TGF-β signaling [27]. Thus, decorin A-induced activation of autophagy might play a vital role in the inhibition of cell migration and proliferation in PC cells. However, overexpression of decorin B activated TGF-β signaling while there was no impact on apoptosis signaling. Thus, these results showed that due to the loss of a fragment, decorin B could not sequester multiple growth factors and, in fact, promoted cancer cell proliferation and migration (Figures 2, 3). The mechanism was shown in Figure 6. Further studies are required to investigate the mechanism of decorin B-induced PC progression.

## CONCLUSIONS

Here, significantly upregulated levels of decorin were found in PC tissues compared with the normal tissues but no significant difference was observed between different pathological stages. These results were further confirmed by the immunoblotting analysis. The results of western blot showed elevated expression of decorin A in para-cancerous tissues than in the cancerous tissues, but the expression of decorin B, C, and D was elevated in the cancerous tissue. The results of the MTT assay and scratch wound healing assay revealed that cell proliferation and migration were enhanced in decorin B-overexpressing cells but inhibited in decorin A-overexpressing cells. Decorin A-overexpression significantly elevated the expression of apoptosis-related genes and decorin B-overexpression elevated proliferation-related genes. These results showed that decorin B played a vital role in the progression of PC.

## MATERIALS AND METHODS

### Survival and expression analysis by GEPIA

We analyzed the expression of decorin as well as its association with the prognosis of PC using Gene Expression Profiling Interactive Analysis (GEPIA) (http://gepia.cancer-pku.cn/index.html). Additionally, the differential expression of decorin between normal and cancerous tissue was visualized using boxplots. We also compared decorin expression during different pathological stages using boxplots with the pathological stage as the variable.

### Immunohistochemical analysis

PC paraffin tissue sections (Shanghai Outdo Biotech (HPanA020PG02)) were dewaxed and rehydrated using xylene and gradient alcohol, respectively, and rinsed twice with PBS-T for 5 min each. Endogenous peroxidase was removed with 3% H_2_O_2_ (in methanol), and antigen repair was performed using EDTA solution (0.05 M, pH 8.0) at 85° C. Next, the tissue sections were cooled to room temperature, rinsed thrice with PBST for 5 min each, and blocked for 2 h with 3 mL of blocking solution (Sangon Biotech, China). The specimen was incubated with the decorin primary antibody (Abcam, Cambridge, MA, USA), followed by the HRP-labeled secondary antibody. The tissue sections were visualized using the DAB color development kit (Solarbio, Beijing, China), and counterstained with hematoxylin and eosin for 2 min, fractionated with HCl for 30 s, blued with tap water, and finally sealed with glycerin gelatin sealing tablets (Solarbio). Nikon E80i microscope (Nikon, Tokyo, Japan) was used to observe and photograph the sections and Image J was used to quantify the signal of decorin for each individual stage (I-III).

### Immunoblotting analysis

The surgically resected cancerous and para-cancerous tissues from PC patients were independently assessed by two experienced pathologists and then stored at -80° C. Next, the clinical and follow-up data of these patients were recorded. The experimental procedure was approved by the Ethics Committee of Jining Medical University. Immunoblotting analysis was performed to determine decorin expression in PC patients. The total protein from the cancerous and the paracancerous tissues was first digested by chondroitinase ABC and then combined with five volumes of loading buffer (10% w/v SDS, 5% v/v β-mercaptoethanol, 250 mM Tris-HCl pH 6.8, 30% v/v glycerol, 0.02% w/v bromophenol blue), and boiled for 5 min. The total protein samples were loaded on polyacrylamide pre-cast gels and electrophoresed at room temperature.

Post-electrophoresis, the gel was immersed in the transfer buffer (192 mM glycine, 25 mM Tris, 20% methanol, pH 8.3) to transfer the proteins to PVDF membranes (Millipore, Billerica, MA, USA) using a Semi-dry film transfer instrument (Biorad, Hercules, CA, USA) at 20 V for 30 min at room temperature. The membranes were incubated with blocking buffer (Sangon Biotech) for 3 h, followed by overnight incubation with the respective primary antibodies (Abcam) at 4° C. Next, the membranes were washed thrice with PBS-T for 5 min each, incubated with secondary antibodies for 1 h, and then, washed thrice with PBST for 5 min each. Finally, the membranes were visualized by incubation in the DAB color development solution (10 mL PBST, 10 mg DAB, 10 μL H_2_O_2_) for 5 min.

### Cell lines and transfections

The BxPC-3 cells were cultured in RPMI 1640 medium (Biosharp, Hefei, China) supplemented with 10% fetal bovine serum (FBS; Biological Industries, Cromwell, CT, USA), sodium pyruvate (1 mM), streptomycin (100 μg/mL), and penicillin (100 units/mL) (Biosharp) at 37° C in 5% CO^2^.

Total RNA was isolated from BxPC3 cells. The designed primers were used to amplify decorin by RT-PCR, followed by the construction of the expression vector pcDNA3.1-DNC. The Xfect Transfection reagent (Takara, Osaka, Japan) was used for transfection. PC cells (1 × 10^5^ cells/well) were plated in a 6-well plate, and 12 hours later, Xfect polymer was incubated with pcDNA3.1-DNA for 10 min and the mixture was added dropwise to the respective wells. After 4 h incubation, the serum-containing medium was added. The pcDNA3.1 empty vector was used as negative control.

### MTT cell viability assay

PC cells (2 × 10^3^ cells/well) were seeded in a 96-well plate. After 24 h, the cells were transfected with decorin A and decorin B. The methyl thiazolyl tetrazolium (MTT) assay (Sangon Biotech) was performed at 48 h post-transfection. Specifically, the cells were incubated with the MTT solution (5 mg/mL; 10 μL) for 4 h at 37° C, followed by the addition of Formazan Solubilization Solution (solution C; 100 μL). The 96-well plate was shaken for 10 minutes to dissolve the formazan dye. A multifunction microplate reader (Biorad) was used to determine the absorbance at 570 nm, with viability calculated as follows: (transfected group−blank) / (control−blank) * 100%.

### Scratch wound healing assay

PC cells (1 × 10^5^ cells/well) were seeded in a 6-well plate. After 24 h, the cells were transfected with DCNA and DCNB. After 48 h of incubation, a 200 μL pipette tip was used to scratch the cells, and the cell debris was discarded by washing thrice with PBS. The cells were incubated in the serum-free growth medium, and the images were captured at 0 h and 48 h.

### RT-PCR

Post-transfection with decorin A and decorin B, RNAiso Plus (Takara) was used to isolate total RNA from the PC cells following the manufacturer’s guidelines. Next, the Prime Script RT reagent kit (Takara) was used to reverse transcribe the RNA to cDNA. Semi-quantitative RT PCR was used to confirm the expression of decorin A and B after transfected while qRT-PCR was used to determine the level of expression of cell proliferation-related genes (PCNA, cyclin A, cyclin B, and cyclin D) and apoptosis-related genes (BCL2, BAX, and p53) using the SYBR Premix Ex Tap reagent kit (Takara, China) with β-actin as the internal reference. The -ΔΔCq method was used to calculate the expression level of target genes.

### Statistical analysis

All experiments were performed at four times with three replicates each time and the data are presented as the mean ± SEM. Significant differences among means were tested by one-way ANOVA (analysis of variance) followed by Duncan’s multiple comparison procedure using GrapPad Prism 8 at a significance level of *p<0.05 and **p <0.01.

### Availability of data and materials

The datasets supporting the conclusions of this article are within the paper.

### Ethics approval

The experimental procedure was approved by the Ethics Committee of Jining Medical University.
